# An Important Paper for July

**Published:** 1893-06

**Authors:** 


AN IMPORTANT PAPER FOR JULY.—Our July number, which begins Vol. 9, will contain a valuable contribution to the literature of Forensic medicine from the pen of the able and distinguished alienist and neurologist, Dr. D. R. Wallace, of Waco. It will be a write up of the famous "Newburg Case," which created so much excitement in Johnson county recently, a case where an old lady of good character, and kind and affectionate disposition, gently took her three little sleeping grand-children out in to the yard and cut their throats. The write up will contain the expert testimony of Dr. Wallace and Dr. T. C. Osborn. Orders for extra copies should be sent in at once, as the edition will be limited.

